# *Culex quinquefasciatus* Holobiont: A Fungal Metagenomic Approach

**DOI:** 10.3389/ffunb.2022.918052

**Published:** 2022-08-02

**Authors:** Guillermo A. M. Flores, Rocio P. Lopez, Carolina S. Cerrudo, V. Fabiana Consolo, Corina M. Berón

**Affiliations:** ^1^ Instituto de Investigaciones en Biodiversidad y Biotecnología (INBIOTEC) - Consejo Nacional de Investigaciones Científicas y Técnicas (CONICET) and Fundación para Investigaciones Biológicas Aplicadas (FIBA), Mar del Plata, Buenos Aires, Argentina; ^2^ Laboratorio de Ingeniería Genética y Biología Celular y Molecular (LIGBCM), Area Virosis de Insectos (AVI), Departamento Ciencia y Tecnología, Universidad Nacional de Quilmes and CONICET, Bernal, Argentina

**Keywords:** mosquito, *Culex quinquefasciatus*, mycobiota, metagenomics, ITS2

## Abstract

Microorganisms associated with mosquitoes have fundamental roles, not only in their nutrition, but also in physiological and immunological processes, and in their adaptation to the environment as well. Studies on mosquito hologenomes have increased significantly during the last years, achieving important advances in the characterization of the “core bacteriome” of some species of health importance. However, the fungal mycobiome has not been exhaustively researched, especially throughout the life cycle of some hematophagous mosquito species. In this work, the diversity and composition of fungal communities in different developmental stages, sexes, and adult nutrition of *Culex quinquefasciatus* reared on laboratory conditions were characterized, using internal transcribed spacer high throughput amplicon sequencing. Larvae presented a higher fungal richness, while sucrose-fed males and females showed a similar diversity between them. Blood-fed females presented few operational taxonomic units with an even distribution. Results are consistent with the reduction of larval microbiota after molting, observed for the bacterial microbiome in other mosquito species. The filamentous Ascomycota *Penicillium polonicum* and *Cladosporium* sp. were present in all stages of the mosquitoes; in addition, the presence of yeasts in the insects or their subsequent colonization associated with their diet is also discussed. These results suggest that some species of fungi could be essential for the nutrition and development of mosquitoes throughout their life cycle.

## Introduction

Studies on hematophagous mosquitoes that feed on human blood are central in public health since these insects are capable of vectorizing a variety of pathogens, many of them potentially deadly for more than half of the world’s population (WHO, 2020). In particular, *Culex quinquefasciatus* is widely distributed in the tropical and subtropical areas in almost all the world, having an important role in the transmission of lymphatic filariasis, West Nile virus, Saint Louis encephalitis virus, the Japanese encephalitis virus (JEV), and other pathogens important to wildlife and human health (Vinogradova, 2000; Becker et al., 2020; Harvey-Samuel et al., 2021).

In recent years there has been an increase in research on mosquito-microbiota interaction, highlighting the role of some microorganisms in mechanisms such as the exploitation of nutrients, obtaining metabolites with physiological or immunological functions, resulting in great adaptability to different environments, in defense against stress agents, and in vector competence, as well (Dong et al., 2009; Dennison et al., 2014; Guégan et al., 2018). It has been demonstrated that the same bacterium can affect distinct mosquito populations in different ways. For instance, the endosymbiont bacteria *Wolbachia*, can modify the pathogen transmission of agents like dengue virus in *Aedes aegypti* lines artificially carrying it (Hoffmann et al., 2011), or the human malaria parasite, *Plasmodium falciparum*, in *Anopheles stephensi* (Bian et al., 2013). However, in natural mosquito populations, the presence of *Wolbachia* can be beneficial for their efficient development, affecting its susceptibility against some mosquito pathogens (Díaz-Nieto et al., 2021; Gil et al., 2021). It has also been seen that the density of intracellular *Wolbachia* has been found to be higher in insecticide resistant mosquito populations (Duron et al., 2006). This clearly shows that the interactions can be multiple, depending on the microorganism-host relationship, type and density of the microorganisms, interactions with the environment, insect nutrition and many other factors, in extremely complex systems. Know and characterize the mosquito holobiont, identifying the ecological community of symbiotic, pathogenic and commensal microorganisms, associated with the mosquitoes body and the assembly of genetic information between them, will allow to understand the complex and coordinated co-evolution of these forms of life (Guerrero et al., 2013; Guégan et al., 2018). On the other hand, it permits the manipulation of the microbiota to control mosquitoes or the transmission of the etiological agents that they vector (Ricci et al., 2011; Huang et al., 2020; Singh and Das, 2020).

At least part of the microorganisms that colonize mosquitoes are acquired during their immature stages in their aquatic habitat, where the larval stages filter organic matter in suspension (Zouache et al., 2022). The microbiota of mosquitoes reared in natural habitats is highly variable and can vary according to their geographic origin, ecological niche, species, sex, and food source (Yun et al., 2014; Coon et al., 2016). At the same time, the microbial density can be influenced by the type of diet ingested by the insect, which depends directly on the stage of development in which it is found and its sex. As it has been shown, the microbiota composition and distribution in *Ae. aegypti* midgut differs from blood digestion (Gusmão et al., 2010); and in *Aedes albopictus* differences in the active microbiota between males and females are identified after being fed on fructose solution (Guégan et al., 2020b). Despite this variability, it has been determined, at least for bacteria and fungi, that they generally contain a core microbiome dominated by a small number of taxa (Guégan et al., 2018; Luis et al., 2019).

Although progress has been made in understanding the mosquito holobiont, its fungal composition is not known in detail. In a few *Aedes* and *Culex* species, Ascomycota and Basidiomycota are detected, including yeasts and filamentous species, with high prevalence of some particular fungus (Malassigné et al., 2020). In this context, some yeasts, including *Wickerhamomyces anomalus* (*Pichia anomala*) have been associated with *An. stephensi*, *Anopheles gambiae*, *Ae. aegypti* and *Ae. albopictus* and proposed as promising species to implement in methods for the control of mosquito-borne diseases (Ricci et al., 2011). Most recently, fungal microbiota communities, associated with some mosquitoes of the *Culex* and *Aedes* genera, were identified through next generation sequencing (Luis et al., 2019; Guégan et al., 2020a). However, the understanding about fungal diversity in mosquitoes and its relationship with other symbiotic microorganisms is very scarce. Therefore, the aim of this work was to identify the fungal community in the *Cx. quinquefasciatus* holobiont from insectary population throughout the mosquito life cycle, comparing the different stages of development, between the sexes in adults, taking into account the nutritional source between males, females fed on sucrose and females fed on vertebrate blood, using high-throughput amplicon sequencing of the fungal internal transcribed spacer (ITS2) fragment.

## Materials and Methods

### Ethics Statement

Protocols for blood feeding mosquitoes on mice were reviewed and approved by the Animal Experimental Committee at the Faculty of Exact and Natural Sciences, Mar del Plata University (Institutional Committee on Care and Use of Experimental Animals (CICUAL) N° 2555-04-14). Mice were handled in strict accordance with National Health Service and Food Quality (SENASA) guidelines (Argentina) following the 2011 revised form of The Guide for the Care and Use of Laboratory Animals published by the U.S. National Institutes of Health.

### Mosquito Rearing and Collection

Larvae and adults of *Cx. quinquefasciatus* were obtained from the insectary of the Biological Control Laboratory of the INBIOTEC-CONICET, FIBA (Argentina). Insects were reared at standard temperature and relative humidity conditions (24°C, 80 ± 5%) and a 12 h light: 12 h dark photoperiod. Larvae were raised in dechlorinated water and fed with commercial fish food (Shulet Carassius), male and female adults were provided with 20% (w/v) sucrose solution and gravid females were allowed to feed blood from mice. Adults were killed by freezing and wings and legs were removed under Nikon SMZ800 stereoscope.

### Mosquito DNA Extraction

A total of 80 individuals (20 insects for each group) were used for the total genomic DNA extraction: 1) third instar larvae 2) sucrose fed male adults 3) sucrose-fed female adults and 4) mice-blood-fed female adults. All sucrose solution-fed adults were processed at 48 h post emergence, and blood-fed females were processed 48 h post blood-feeding (96 h post emergence, after blood digestion). Each insect pool (in duplicates) was surface sterilized with 70% ethanol for 1 min, followed by 3 to 4 washes with sterile phosphate-buffered saline solution (PBS), for 1 min each time. Samples were frozen on liquid nitrogen and homogenized with the DNA extraction kit lysis buffer (DNeasy Blood & Tissue kit, QIAGEN), using glass beads and a teflon tip in a mechanical cell lyser equipment. This process was carried out in four cycles of freezing/drilling, 1 min each. The homogenates were incubated with 2 mg/ml of proteinase K during 3 h for protein degradation, then centrifuged at 12000 rpm. After that, the lysate was transferred to the kit column, following the DNA extraction, according to the manufacturer’s protocol.

### Metagenome Amplicon Sequencing

Metagenome amplicon sequencing of the fungal ribosomal RNA gene was performed using gDNA isolated from each sample. Extracted DNA was quantified by picogreen method using Victor 3 fluorometry (Invitrogen, Waltham, MA, USA) and quality condition assessed by gel electrophoresis method. From each mosquito group, sequencing libraries for nuclear ribosomal internal transcribed spacer (ITS2) were constructed. ITS3 (5´- GCATCGATGAAGAACGCAGC -3´) and ITS4 (5´- TCCTCCGCTTATTGATATGC -3´) primers (Bellemain et al., 2010) were used on an Illumina Miseq 300 bp PE platform, in accordance with Illumina 18S rRNA metagenomic sequencing library protocols (Macrogen Inc., Korea). 

### Sequence Analysis and Taxonomic Assignment

The quality of the paired-end reads in .fastq files was initially checked by FastQC. Further, with a combination of UPARSE-pipeline (Edgar, 2013) and mothur software, applying quality and quimera removing filters, with a minimum length overlap of 50, a maximum number of mismatches in the overlap region of 12; the reads longer than 250 bp with an expected error < 1 were clustered into operational taxonomic units (OTUs) at a level of 97% similarity. Taxonomic assignment was performed on representative sequences from each OTU using massBLASTer and SH mappings of the UNITE database (Nilsson et al., 2019). Unclassified OTUs were taxonomically identified by performing Blast searches against the GenBank standard database, Nucleotide collection (nr/nt), (**Supplementary Figure S1**).

### Bioinformatic Analysis

To analyze sequencing depth in each mosquito group, rarefaction curves with vegan package 2.5 (Oksanen et al., 2020) were constructed. Alpha-diversity indexes to describe the communities diversity were calculated and measured in two categories for each group: richness and diversity. Margalef, Fisher_alpha, Shannon, Inverse Simpson index (InvSimpson) and Sorensen’s indexes were computed using mothur (Schloss et al., 2009) and vegan package 2.5, with a randomization of 100 runs and sampling with replacement. Thus, the Shannon index quantifies species diversity for each collected sample, combining measures of richness and evenness. Margalef index is a specific richness index, Fisher_alpha index applied to richness estimation, is theoretically independent of sample size, and InvSimpson represent the inverse of the probability that two randomly selected individuals in the habitat will belong to the same species. Relative abundance was calculated, and identified taxa were analyzed with Krona (Ondov et al., 2011).

Fungal community composition from mosquito instar populations (ß-diversity) were primarily analyzed using non-metric multidimensional scaling (NMDS) plot by R software v.4.1.2. (RC Team, 2021). The distance matrix was computed based on Bray-Curtis and Euclidean distance analysis with vegan package 2.5, these distances were used to construct NMDS and principal component analysis (PCA) respectively. The PCA was performed in the Galaxy web platform usegalaxy.org (Afgan et al., 2018); with a combination of ggplot2 and ggfortify. Plots were made on Graphpad Prism 8.0.1.

## Results

### Fungal Species Diversity in *Cx. quinquefasciatus*

#### Mosquito Fungal Alpha Diversity

In order to explore the fungal diversity throughout the *Cx. quinquefasciatus* development, amplicons of the ITS2, from the ribosomal DNA region, were sequenced. A total of 1,509,358 paired-end reads were obtained from gDNA of larvae, males and females fed on sucrose, and females fed on mice blood (**Supplementary Table S1**). After quality filtering, 34,603 reads were assigned to 55 fungal OTUs at 97% identity (**Supplementary Table S2** and **Supplementary Data Sheet 1**). Rarefaction curves showed that the sequencing depth was sufficient to cover the fungal diversity observed in the mosquito groups (**Supplementary Figure S2**). Only for the blood-fed females sample the plotted curve did not reach a plateau, indicating that the fungal community was not well represented.

**Figure 1:**
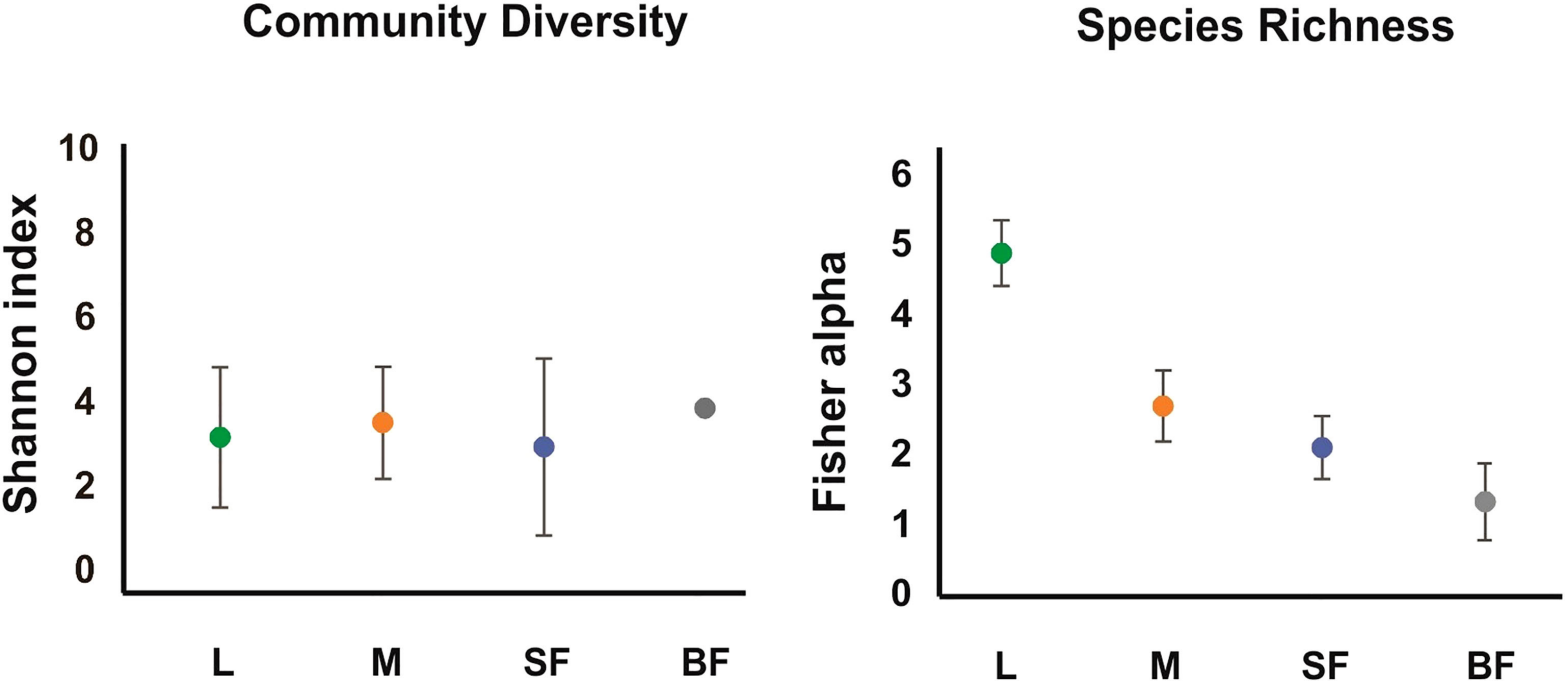
Alpha diversity of the fungal communities along *Cx. quinquefasciatus* development. The alpha-diversity was estimated with diverse indexes for different instars. The Fisher_alpha, and Shannon indices are represented in the graphs. L, larvae; M, sucrose-fed male; SF, sucrose-fed females; BF, blood-fed females. The error bars represent the 95% confidence interval, obtained with a randomization of 100 runs and sampling with replacement.

Diversity estimators, Shannon and InvSimpson indices (**Table 1** and **Figure 1**) which measure the richness and homogeneity of the samples, considering the evenness of the species relative abundance, showed similar values for all mosquito groups. Neither of the two indices allow to obtain a comparable diversity value between the mosquito stages. This is because the stages with the highest number of OTUs present heterogeneity and dominance of one species, while the sample with the least number of OTUs (blood-fed females) presents homogeneity (it has few species, but each one has similar relative abundance).

**Table 1:** Summary of community richness and alpha diversity per *Cx. quinquefasciatus* instars.

Sample	OTUs^a^	Reads	Dominance^b^	Margalef	Fisher_alpha	Shannon	InvSimpson	Good´s coverage^c^
Larvae	41	21266	0.370	4.014	4.89 (4.42-5.35)	3.04 (1.37-4.71)	2.06 (0.85-3.27)	0.9981
Males	22	9169	0.711	2.301	2.71 (2.20-3.21)	3.39 (2.06-4.72)	2.23 (1.25-3.21)	0.9976
Sugar-fed female	16	4113	0.750	1.802	2.11 (1.66-2.56)	2.99 (0.89-5.09)	2.29 (0.05-4.52)	0.9961
Blood-fed female	5	55	0.232	0.998	1.34 (0.79-1.88)	3.73 (3.73-3.73)	2.38 (2.38-2.38)	0.9090

**^a^** OTUs: Operational Taxonomic Units.

**^b^** Dominance: defined as pi^2^, where p_i_ is the relative abundance of OTU_i_.

**^c^** Good’s coverage: C= 1-(F_1_/N), where F_1_ is the number of singletons OTUs and N is the number of reads in the sample (which is the sum of counts from all OTUs).

The average value for each index is indicated and the confidence interval in parentheses.

On the other hand, to evaluate species richness in the different mosquito stages Margalef and Fisher_alpha indexes were calculated (**Table 1** and **Figure 1**), observing that the larvae present greater richness than the adult stages. Indeed, fungal species richness seems to decrease with mosquito development. 41 fungal OTUs were identified in the larval stage (74.54% of the total OTUs from all samples), in which 27 were exclusively in this stage. 6 and 3 unique fungal OTUs were identified in adult males and females fed on sucrose solution respectively, while 2 were detected only in blood-fed females. Furthermore, although there seem to be no major differences between males and females, a significant decrease in species richness was observed once the adult females were fed on blood.

#### Fungal Beta Diversity

**Figure 2:**
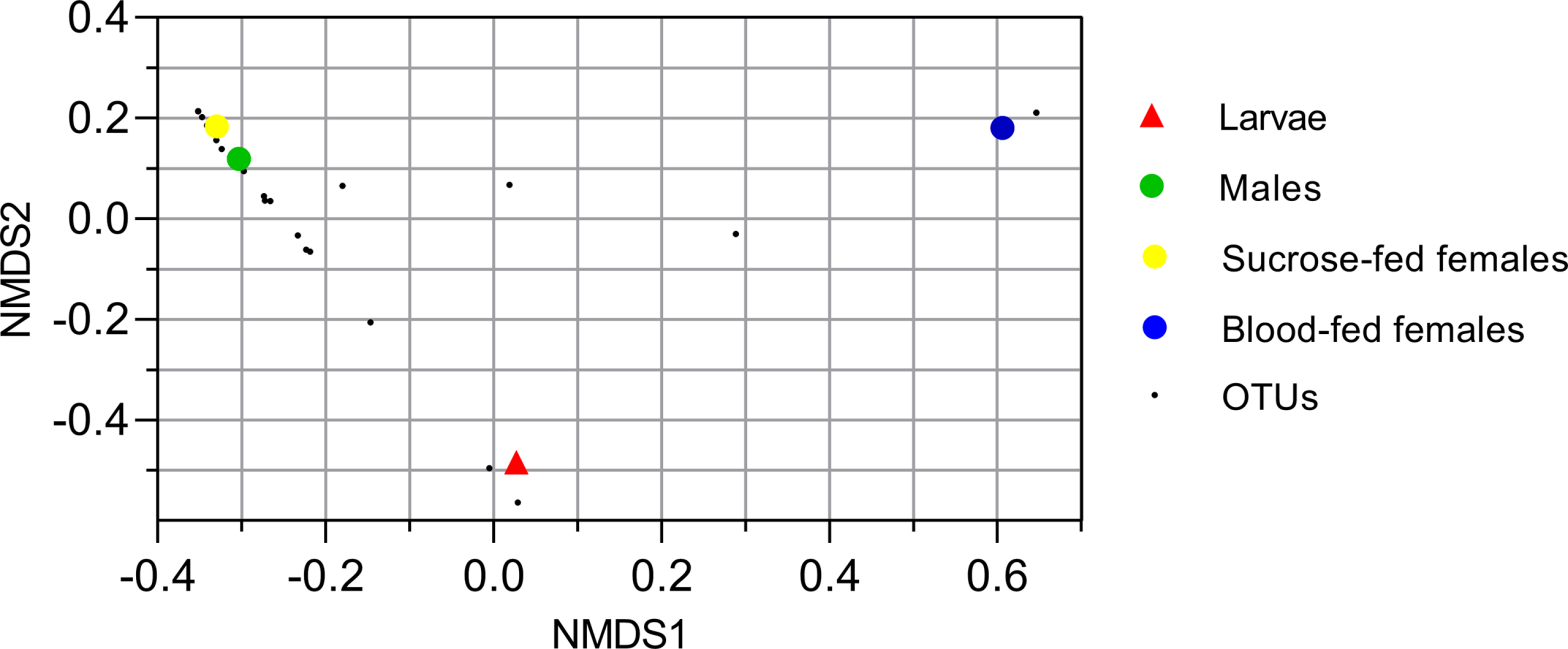
Nonmetric multidimensional scale ordination plot of groups based on Bray-Curtis dissimilarity matrix. Fungal operational taxonomic units (OTUs) are represented with small black points. Larvae, male and sucrose-fed females are in duplicates.

**Figure 3:**
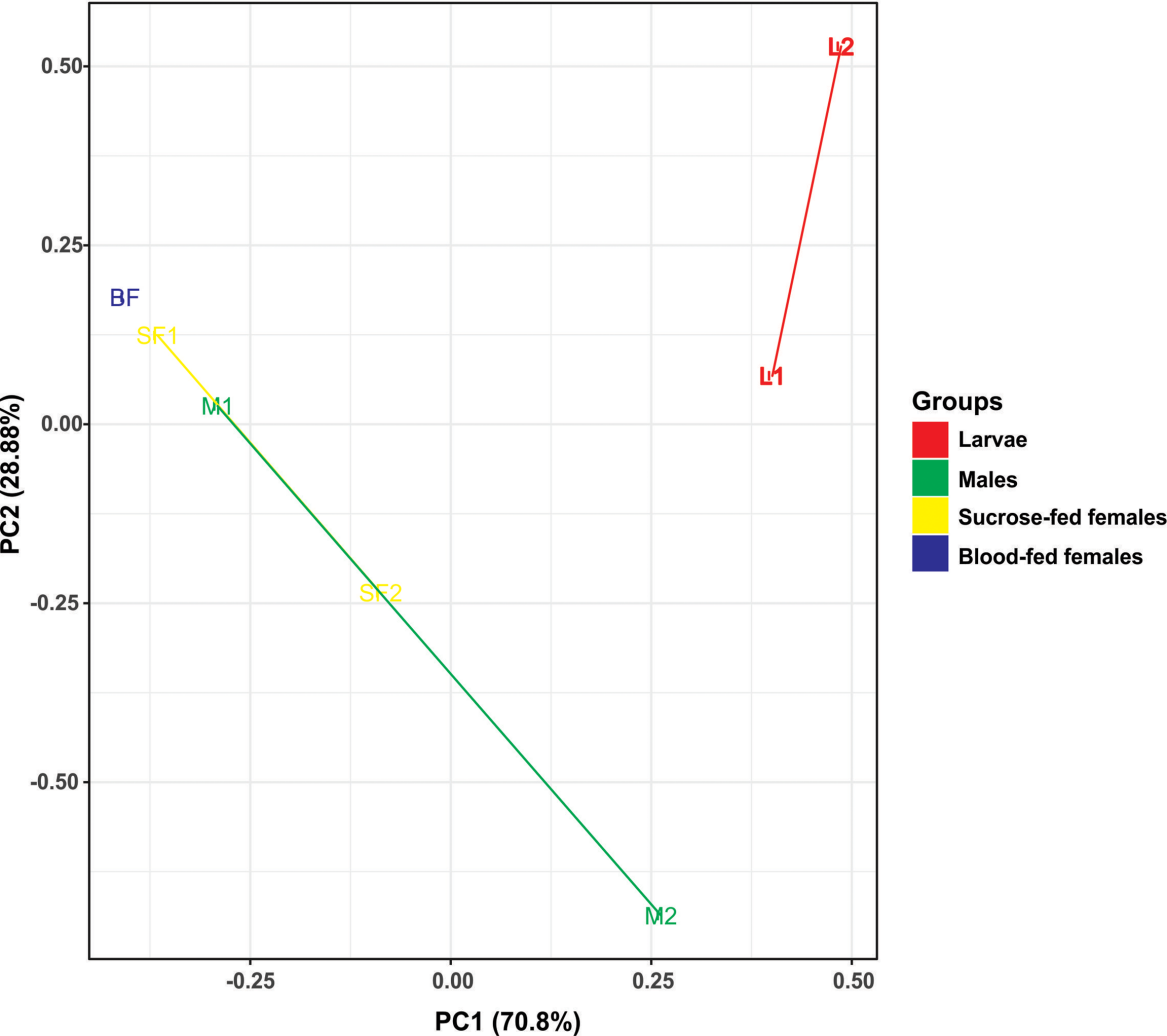
Principal component analysis (PCA) based on the Euclidean distances for fungal communities from samples along the *Cx. quinquefasciatus* development. There are two distinct groups, adults and larvae, using two principal components (PC1 and PC2).

To analyze the similarity between fungal communities in different mosquito instars and sex, throughout the life cycle of *Cx. quinquefasciatu*s laboratory line, NMDS and PCA were performed. The general comparison allowed us to observe that there are distinct clusters that differentially grouped the fungal communities of larval and adult samples (**Figures 2**, **3**). Moreover, samples from the adult stage present a similar fungal community regardless of whether they are male or female. These observations are supported by a high similarity value of Sorensen index between males and sucrose-fed females (0.63) and lower values for larvae and adults (0.13 - 0.41), blood-fed females and the other groups (0.13 - 0.22) (**Supplementary Table 3**).

### Fungal Communities Composition in *Cx. quinquefasciatus*

The composition of the *Cx. quinquefasciatus* fungal communities at different taxonomic levels was analyzed, assigning the taxonomic nomenclature of each OTU through the UNITE database. The identified OTUs corresponded to 4 phyla, Ascomycota (58%), Basidiomycota (34.5%) Mucoromycota (0.036%) and Morteriellomycota (0.018%), although other 2 OTUs were unidentified. Even though, the taxonomic affiliation of the sequences was able to identify fungal species level, in some cases, only phylum was reached.

**Figure 4:**
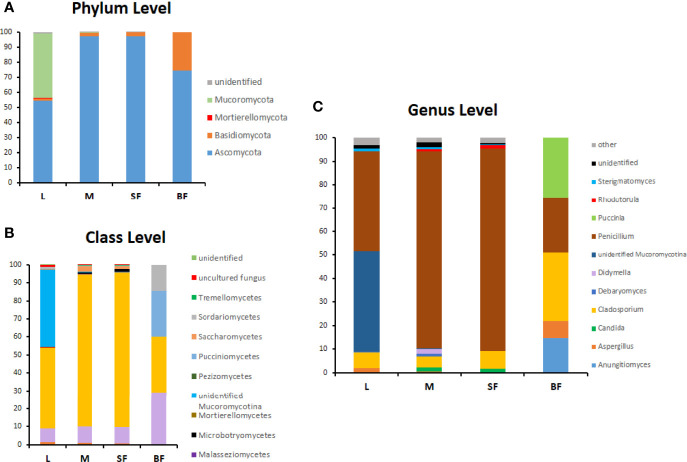
Relative abundance (%) of fungi along *Cx. quinquefasciatus* development at the phylum **(A)**, class **(B)** and genus level **(C)**. OTUs with values >1% were grouped under other genera in panel **(C)**.

At the phylum level, Ascomycota was the most abundant in all stages, representing more than 90% of the sequences in sucrose-fed males and females (**Figure 4**). In these groups more than 80% of the Ascomycota abundance was due to the presence of *Penicillium polonicum* (Eurotiomycetes). This fungus was detected in all stages with a relative abundance between 24 to 86% (**Table 2**). Particularly, it was predominant in sucrose-fed adults (84.10% males and 86.21% females); however, in larvae the relative abundance was 42.8% and 23.64% in blood-fed females. In comparison, the phylum Basidiomycota presented a low percentage in all stages (up to 2.4%), except for blood-fed females in which it was represented by 25.45% of their total fungal sequences, composed of a single OTU, corresponding to the Pucciniomycete *Puccinia sorghi*, found only in this stage. The remaining sequences, corresponding to Basidiomycota, were identified as subphylum *Agaricomycotina*, with classes Agaricomycetes and Tremellomycetes, including filamentous fungi (46.9%) and yeasts (53.07%). In particular, yeasts or yeast-like sequences detected in *Cx. quinquefasciatus*, belong to the sub-phyla *Pucciniomycotina* and *Agaricomycotina* (Basidiomycota), *Pezizomycotina* and *Saccharomycotina* (Ascomycota).

**Table 2:** Fungal classified OTUs identified throughout *Cx. quinquefasciatus* development.

Phylum	Fungal species	Relative abundance (%)			
		L	M	SF	BF
**Filamentous fungi**					
Ascomycota	*Penicillium polonicum*	42.8	84.1	86.21	23.64
Ascomycota	*Cladosporium* sp	6.71	4.67	7.61	29.09
Ascomycota	*Didymella exigua*	–	2.17	0.12	–
Ascomycota	*Anungitiomyces stellenboschiensis*	–	–	–	14.55
Ascomycota	*Aspergillus penicillioides*	1.71	0.32	–	7.27
Basidiomycota	*Puccinia sorghi*	–	–	–	25.45
Mucoromycota	unidentified *Mucoromycotina*	42.63	0.24	–	–
**Yeast/Yeast-like fungi**					
Ascomycota	*Candida parapsilosis*	0.22	1.63	1.48	–
Ascomycota	*Debaryomyces prosopidis*	0.07	1.1	–	–
Basidiomycota	*Rhodotorula mucilaginosa*	–	0.85	1.39	–
Basidiomycota	*Sterigmatomyces halophilus*	1.06	0.8	0.24	–

L, larvae; M, sucrose-fed male; SF, sucrose-fed females; BF, blood-fed females. Each library was constructed from groups of 20 individuals. OTUs with more than 1% of relative abundance in any stage were included.

The larval stage showed the greatest diversity of fungal species since 41 OTUs were determined, with dominance of *Penicillium* and *Mucoromycotina* (approximately 43% abundance each). 7 OTUs (12.7%) are shared by three groups (larvae, males, and sucrose-fed females), belonging to the following Ascomycota orders: *Agaricostilbales*, *Saccharomycetales*, *Capnodiales*, *Hypocreales*, *Pleosporales*, *Capnodiales*, *Malasseziales* and 1 OTU identified as member of the *Aspergillaceae* family is shared by larvae, males and blood-fed females. Sucrose- and blood-fed females are the 2 groups that share the minor amount of OTUs, in which *Penicillium* and *Cladosporium* were present in both of them. Sucrose-fed females and males share 3 OTUs of *Didymella*, *Rhodotorula* and *Cutaneotrichosporon* yeast genera; while larvae and males from *Mucoromycotina, Debaryomyces* and *Aspergillus*.

**Figure 5:**
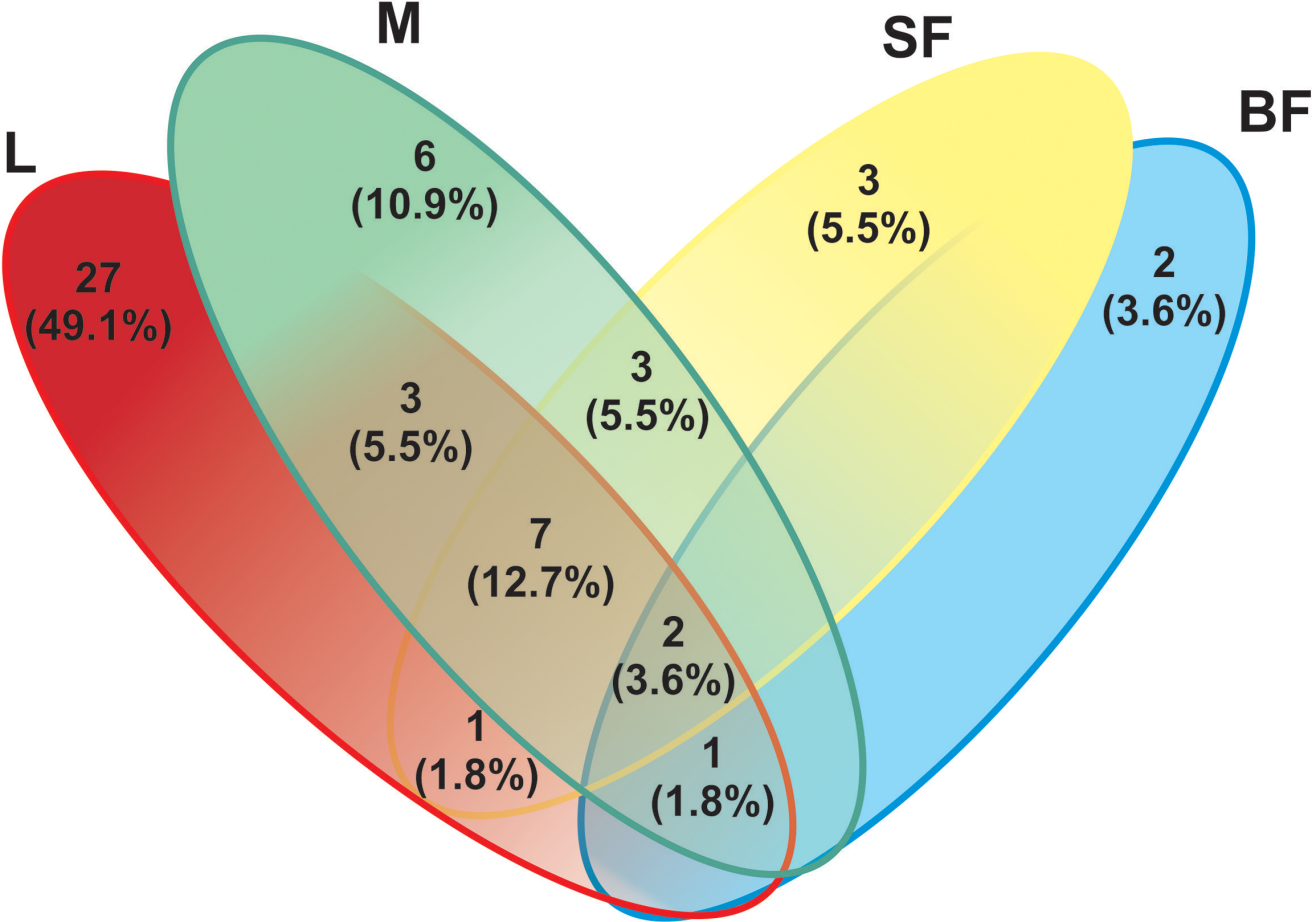
Venn-diagram depicts the number of fungal ITS Operational Taxonomic Units (OTUs) overlap and non-overlap between *Cx. quinquefasciatus* instars. A total of 55 fungal OTUs (based on the sequencing of the ITS-2 regions) were detected. L, larvae; M, male; SF, sucrose-fed females; and BF, blood-fed females.

Finally, the blood-fed females present a lower richness but with an even distribution of the OTUs (*Penicillium*, *Cladosporium* and *Puccinia* which were present in a proportion of nearly 25% each one), sharing fewer OTUs with the other groups. OTUs shared in all mosquito stages are shown in **Figure 5** and **Supplementary Table 2**.

## Discussion

Research on the mosquito microbiome has increased significantly during the last years, highlighting its role in the biology of mosquitoes and its potential as agents for the development of control strategies for diseases transmitted by them (Dada et al., 2021). In this context, species of microorganisms associated with the mosquito holobiont that affect its nutrition, development and susceptibility to pathogens, and its vectorial competence as well, have been described through the use of culture-dependent and independent techniques (Dennison et al., 2014; Duguma et al., 2015; Guégan et al., 2018). In particular, the main efforts have been made on bacteria, while few studies have described the whole fungal communities associated with these insects. In this work, the fungal composition of *Cx. quinquefasciatus* mosquito larvae and adults, including female blood-fed, reared in laboratory conditions, were characterized through a metagenomic approach. The highest fungal richness was observed in larval stages, such as in mosquitoes reared in natural sites, even though our population was bred under controlled laboratory conditions. This result agrees with Zouache et al. (2022), who suggested that the mosquito microbiota could be acquired during their feeding habit of filtering and grazing. In particular, the diversity and composition of mosquito mycobiota has been determined, at least partially, by the diversity and composition of microorganisms in their habitats (Thongsripong et al., 2017), as it has been observed in *Ae. albopictus* larval guts (Tawidian et al., 2021). Decrease in fungal diversity between *Cx. quinquefasciatus* larvae and all adult samples could be explained by the effects of the metamorphosis process, in which mosquito larvae suffer drastic morphological changes that reduce their microbial abundance, especially by the egestion of the last larval food bolus and then of the midgut meconium in newly emerged adults (Moll et al., 2001) (**Table 1**).

It has been reported that fungal microbiome communities of several mosquito species are composed mainly by the phylum Ascomycota (especially *Pezizomycotina*), and Basidiomycota in a lower proportion (Muturi et al., 2016; Thongsripong et al., 2017; Luis et al., 2019; Zouache et al., 2022). Thus, in our work and according to these studies, diversity, and phyla distribution observed was dominated by Ascomycota fungi. Among the fungal OTUs, 2 of them were widespread in almost all individuals. The most abundant species was *P. polonicum*, a common filamentous saprophytic fungus, which was found in all mosquito instars, mostly in adults as male and sucrose-fed females (**Table 2**). This fungal genus was found associated with mosquitoes, in many studies including culture dependent and independent approaches (Pereira et al., 2009; Muturi et al., 2016; Luis et al., 2019; Zouache et al., 2022). Although this saprophyte could be maintained throughout the mosquito development, the role in its life cycle is unknown. It has been pointed out that this fungus produces several metabolites that can be used in insect control (Ragavendran et al., 2019). In this way a *Penicillium citrinum* strain showed high mortality rates against *Cx. quinquefasciatus* larvae, due partially to patulin, a metabolite secreted by this fungus (Maketon et al., 2014). Taken together, these observations suggest that *P. polonicum* as other related saprobes could have a role that is still unknown in mosquitoes and may be a candidate for future studies in *Cx. quinquefasciatus* control strategies.

Additionally, we found *Cladosporium* sp., another filamentous ascomycete in all mosquito stages. This genus was found in *Cx. quinquefasciatus* populations collected from natural habitats, and in other important mosquito vectors like *Cx. pipiens* and *Ae. albopictus* as well, suggesting that its presence in adult females may be due to its occurrence in mosquito environments (Chandler et al., 2015; Thongsripong et al., 2017; Luis et al., 2019). *Cladosporium* is one of the commonest genera in indoor environments, and under certain conditions some species become predominant (Bensch et al., 2018). Maybe *Cx. quinquefasciatus* could have acquired it from the natural environment, and later passed on to its progeny, keeping it throughout development and in its breeding site in our laboratory conditions.

Another identified filamentous fungi group belonged to the phylum Mucoromycota and subphylum *Morteriellomycotina* which was present in a low percentage (**Table 2**). It was a dominant on larval stage (approximately 43% abundance). It was not possible to obtain deeper taxonomic information about the OTU of this group. Thus, these fungi are reported as a diverse group but for the majority of taxa, their ecological role and their geographic distribution remain unknown (Walther et al., 2019).

Yeast or yeast-like Ascomycetes and Basidiomycetes were also identified as an important part of the mycobiome in our mosquito population, although in low total relative abundance (less than 5%). This group included species like *Candida parapsilosis*, *Sterigmatomyces halophilus* and *Debaryomyces prosopidis* in larvae and sucrose-fed adults, while *Rhodotorula mucilaginosa* was only found in sucrose-fed adults (**Table 2**). *Candida* sp. was reported as part of the “core microbiome” in *Ae. albopictus* (Luis et al., 2019) and *C. parapsilosis* stably associated with several mosquito species, including *Cx. quinquefasciatus* (Bozic et al., 2017). Also, Steyn et al. (2016) were able to isolate, through culturable methods, not only *Candida* spp. but also *Rhodotorula* spp. and *Hannaella* sp. in *Culex* mosquitoes, indicating that adults harbored much less yeasts than larvae, suggesting a diminished transmission to the mature stage. This data is consistent with our results, being this the first report of *S. halophilus* and *D. prosopidis* in mosquitoes.

In nature, fungal colonization of the mosquito body probably may occur during the contact and ingestion of plant sugar products. Particularly, in adult mosquitoes fed on nectar and captured in the field, many yeast genera, also present on flowers, were detected (Luis et al., 2019). In this work, laboratory-raised adults were fed on sucrose solution, but diluted in non-sterile dechlorinated water. Novak Babič et al. (2016) studying tap water fungal communities identified different yeast and yeast-like as common species in drinking water. Among them *R. mucilaginosa*, *C. parapsilosis* and *Aureobasidium* sp. occurred in *Cx. quinquefasciatus* adults, probably explaining the presence of certain fungal species in mature stages in this work. Interestingly, *R. mucilaginosa* was only found in sugar-fed males and females. Moreover, some filamentous fungi like *Cladosporium* and *Aspergillus*, especially metabolically active in males, could have a role in fructose digestion, as analyzed in *Ae. albopictus* adult (Guégan et al., 2020b). The presence of the same genera in our laboratory mosquito line, indicates that adult feeding habits could be contributing not only to fungal recolonization after adult emergence, but also to incorporation of fungal symbionts involved in nutrition.

*Cx. quinquefasciatus* females storage nutrients after blood ingestion, increase their reproductive fitness, since blood digestion confers lipids associated with longevity and proteins provisioning yolk for egg production. The blood meal produces changes in gut physiology, modifying the microbiota relative abundance, well studied in bacterial communities (Wang et al., 2011; Telang and Skinner, 2019). However, there are few reports about the fungal diversity changes in females, as seen for the yeast *Meyerozyma*, where its proliferation was favored in two *Aedes* species after blood ingestion (Muturi et al., 2016). In this study we observed a strong change in OTUs dominance in blood-fed females, compared to the sucrose-fed adults: *P. polonicum* abundance decreased and *Cladosporium* became dominant, besides the fungal community was reduced to 5 OTUs with an equitative distribution between species.

Fungal communities in *Cx. quinquefasciatus* presented significant specific signatures, showing differences in composition across their life stage and among reproductive states. Indeed, beta diversity analyses showed a pattern of sample-related clustering composition in agreement with the specific lifestyle of each developmental stage, indicating the presence of sample-type related mycobiota. Species composing the “core mycobiota” in males and females differ, almost completely, from the larval microbiota, suggesting that striking differences in larval diet, sucrose-based adults, and blood-fed females, can generate changes in the intestinal microbial diversity and composition.

In summary, our results provide information on the fungal composition of mosquitoes reared exclusively under controlled laboratory conditions. We suggest that the described mycobiota may be essential for mosquito survival and could have important implications in their biology and pathogen transmission. Species from the mosquito “core mycobiota” could be considered as potential targets and could open the possibility to further experimental studies which exploit them as potential microorganisms in the development of new biotechnological strategies for the mosquito vector-borne diseases control.

## Data Availability Statement

The data presented in the study are deposited in the NCBI repository, accession number PRJNA828597.

## Author Contributions

GF, RL, VC, and CB designed the research plan. GF, RL, and CC performed the bioinformatics analysis. VC and CB acquired funding and supervised the work. All authors contributed to result analysis, wrote and edited the manuscript, read, and approved the submitted version.

## Funding

This study was supported by Grants of the Consejo Nacional de Investigaciones Científicas y Técnicas (CONICET), PUE 2017-0101 (CB) and PIP 2021-2023 1012 (VC), and Universidad Nacional de Mar del Plata (15/E883 EXA925/19) (CB).

## Conflict of Interest

The authors declare that the research was conducted in the absence of any commercial or financial relationships that could be construed as a potential conflict of interest.

## Publisher’s Note

All claims expressed in this article are solely those of the authors and do not necessarily represent those of their affiliated organizations, or those of the publisher, the editors and the reviewers. Any product that may be evaluated in this article, or claim that may be made by its manufacturer, is not guaranteed or endorsed by the publisher.

## Supplementary Material

Supplementary Figure 1 Algorithm representing the analysis performed in this work. For the fungi molecular identification amplicon sequencing based on Internal Transcribed Spacer (ITS) were used. After sequence processing and analysis, the resulting ITS sequences were compared against a known database and the fungal species were identified. In this work we used several tools according with Gao et al. (2021) following the main steps: quality control (grey box) and data preprocessing (yellow box), clustering of reads in OTUs (blue box) and taxonomic assignment (green box), community characterization (orange box). In the present work we used genomic DNA of larvae, males, females only fed on sucrose solution and females fed on mouse blood for fungal ITS2 amplicon sequencing, with an Illumina Miseq 300 bp paired-end (PE) platform. The databases, the programs and their main parameters used in each step are indicated.

Supplementary Figure 2 Rarefaction curves described for observed OTUs metric among all mosquito groups: L, larvae; M, sucrose-fed male; SF, sucrose-fed females; BF, blood-fed females. The rarefied number of operational taxonomic units (OTUs, with 97% sequence similarity cut-off value) is plotted against the number of reads sampled.

Supplementary Data Sheet 1 Krona plots of *Cx. quinquefasciatus* fungal holobiont identified in each development instar. L, larvae; M, sucrose-fed male; SF, sucrose-fed females; BF, blood-fed females. The results are illustrated in an interactive zooming and multi-layered fungal map obtained by Krona (html format), showing the mean taxonomic distribution and relative abundance of taxa.

Supplementary Table 2 Reads assigned as fungal OTUs at 97% identity from all mosquito stages: L, larvae; M, sucrose-fed male; SF, sucrose-fed females; BF, blood-fed females.
