# Supplementary figures and images for: Culex quinquefasciatus Holobiont: A Fungal Metagenomic Approach
Source: Front Fungal Biol. 2022 Aug 2;3:918052. doi: 10.3389/ffunb.2022.918052 (PMC10512223; doi:10.3389/ffunb.2022.918052)

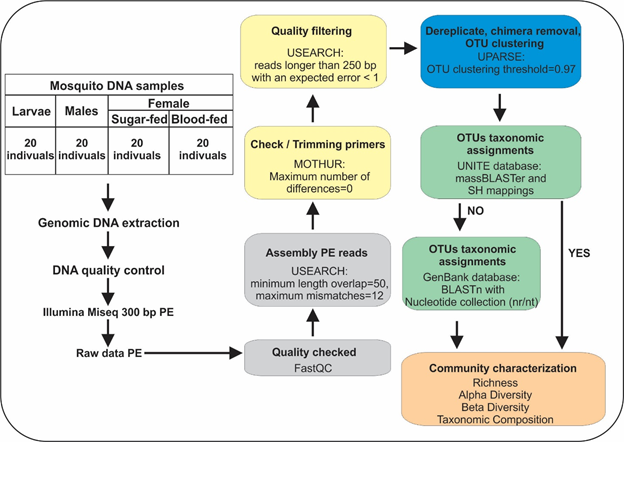

Supplement: Supplementary Figure 1 — Algorithm representing the analysis performed in this work. For the fungi molecular identification amplicon sequencing based on Internal Transcribed Spacer (ITS) were used. After sequence processing and analysis, the resulting ITS sequences were compared against a known database and the fungal species were identified. In this work we used several tools according with Gao et al. (2021) following the main steps: quality control (grey box) and data preprocessing (yellow box), clustering of reads in OTUs (blue box) and taxonomic assignment (green box), community characterization (orange box). In the present work we used genomic DNA of larvae, males, females only fed on sucrose solution and females fed on mouse blood for fungal ITS2 amplicon sequencing, with an Illumina Miseq 300 bp paired-end (PE) platform. The databases, the programs and their main parameters used in each step are indicated. [file Image_1.tif]

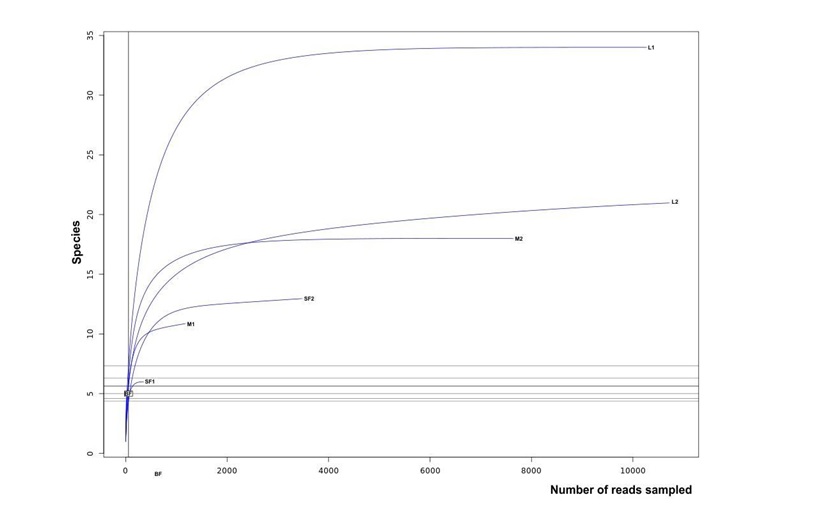

Supplement: Supplementary Figure 2 — Rarefaction curves described for observed OTUs metric among all mosquito groups: L, larvae; M, sucrose-fed male; SF, sucrose-fed females; BF, blood-fed females. The rarefied number of operational taxonomic units (OTUs, with 97% sequence similarity cut-off value) is plotted against the number of reads sampled. [file Image_2.jpeg]
