# Supplementary material for: Culex quinquefasciatus Holobiont: A Fungal Metagenomic Approach
Source: Front Fungal Biol. 2022 Aug 2;3:918052. doi: 10.3389/ffunb.2022.918052 (PMC10512223; doi:10.3389/ffunb.2022.918052)
Supplement: Supplementary Data Sheet 1 — Krona plots of Cx. quinquefasciatus fungal holobiont identified in each development instar. L, larvae; M, sucrose-fed male; SF, sucrose-fed females; BF, blood-fed females. The results are illustrated in an interactive zooming and multi-layered fungal map obtained by Krona (html format), showing the mean taxonomic distribution and relative abundance of taxa. [file DataSheet_1.zip › Supplementary figure S2 (1).html]

Javascript must be enabled to view this page.

magnitude
score


Larvae
Male
Sucrose-fed Female
Blood-fed female

 104
 104
 104
 104

 .992765628052001
 .992554342810641
 .951719688043539
 0

 54.523652779084
 97.3497655142327
 97.3984925844882
 74.5454545454545

 .992238033635187
 .992045709164239
 .995756365451822
 0

 44.9543872848679
 84.5893772494274
 86.2144420131291
 30.9090909090909

 .994769874476988
 1
 1
 0

 44.5640929182733
 84.5893772494274
 86.2144420131291
 30.9090909090909

 .997467553023109
 1
 1
 0

 44.5640929182733
 84.5893772494274
 86.2144420131291
 30.9090909090909

 .997467553023109
 1
 1
 0

 42.7960124141823
 84.0985930853964
 86.2144420131291
 23.6363636363636

 1
 1
 1
 0

 42.7960124141823
 84.0985930853964
 86.2144420131291
 23.6363636363636

 1
 1
 1
 0

 1.76808050409104
 .490784164030974
 0
 7.27272727272727

 .936170212765957
 1
 1
 0

 1.71165240289664
 .316283127931072
 0
 7.27272727272727

 1
 1
 1
 0

 5.64281011943948E-02
 .174501036099902

-1
 1

 .390294366594564

 .686746987951807

 .390294366594564

 .686746987951807

 .21630772124518

 1

 .21630772124518

 1

 .173986645349384

 .297297297297297

 6.11304429605944E-02

-1

 6.11304429605944E-02

 1

 5.17257594281952E-02

 1

 7.6177936612433
 9.2485549132948
 9.23899829807926
 29.0909090909091

 .983950617283951
 .91627358490566
 .955263157894737
 0

 7.18988056051914
 6.87097829643364
 8.24215900802334
 29.0909090909091

 1
 .887301587301587
 .964601769911504
 0

 6.71494404213298
 4.66790271567237
 7.61001701920739
 29.0909090909091

 1
 1
 1
 0

 6.71494404213298
 4.66790271567237
 7.61001701920739
 29.0909090909091

 1
 1
 1
 0

 6.71494404213298
 4.66790271567237
 7.61001701920739
 29.0909090909091

 1
 1
 1
 0

 .315056898335371
 1.42872723306795
 .607828835399951

 1
 1
 .56

 .315056898335371
 1.42872723306795
 .607828835399951

 1
 1
 .56

 .315056898335371
 1.42872723306795
 .607828835399951

 1
 1
 .56

 .159879620050785
 .774348347693314
 2.43131534159981E-02

 1
 0
 0

 .155177278284586

 1

 .155177278284586

 1

 4.70234176619957E-03
 .774348347693314
 2.43131534159981E-02

 1
 0
 0

 4.70234176619957E-03
 .774348347693314
 2.43131534159981E-02

 1
 0
 0

 .427913100724161
 2.37757661686116
 .99683929005592

 .714285714285714
 1
 .878048780487805

 .427913100724161
 .130875777074926
 .87527352297593

 .714285714285714
 1
 1

 .366782657763566
 .130875777074926
 .87527352297593

 1
 1
 1

 .366782657763566
 .130875777074926
 .87527352297593

 1
 1
 1

 6.11304429605944E-02

-1

 6.11304429605944E-02

-1

 0
 2.17035663649253
 .12156576707999

 0
 1
 0

 0
 2.17035663649253
 .12156576707999

 0
 1
 0

 0
 2.17035663649253
 .12156576707999

 0
 1
 0

 0
 7.63442032937071E-02

 0
 1

 0
 7.63442032937071E-02

 0
 1

 0
 7.63442032937071E-02

 0
 1

 .282140505971974
 3.08648707601701
 1.48310235837588

 1
 1
 1

 .282140505971974
 3.08648707601701
 1.48310235837588

 1
 1
 1

 .21630772124518
 1.62504089868034
 1.48310235837588

 1
 1
 1

 .21630772124518
 1.62504089868034
 1.48310235837588

 1
 1
 1

 .21630772124518
 1.62504089868034
 1.48310235837588

 1
 1
 1

 6.58327847267939E-02
 1.10153779038063

 1
 1

 6.58327847267939E-02
 1.10153779038063

 1
 1

 6.58327847267939E-02
 1.10153779038063

 1
 1

 0
 .359908386956048

 0
 1

 0
 .359908386956048

 0
 1

 0
 .359908386956048

 0
 1

 1.0815386062259
 .425346275493511
 .461949914903963
 14.5454545454545

 .939130434782609
 1
 1
 0

 1.0815386062259
 .425346275493511
 .461949914903963

 .939130434782609
 1
 1

 .719458290228534
 .327189442687316
 .461949914903963

 1
 1
 1

 .719458290228534
 .327189442687316
 .461949914903963

 1
 1
 1

 .719458290228534
 .327189442687316
 .461949914903963

 1
 1
 1

 .268033480673375

 1

 .268033480673375

 1

 .268033480673375

 1

 .032916392363397
 9.81568328061948E-02

-1
 1

 .032916392363397
 9.81568328061948E-02

-1
 1

 .032916392363397

-1

 0
 9.81568328061948E-02

 0
 1

 6.11304429605944E-02

 1

 6.11304429605944E-02

 1

 0
 0
 0
 14.5454545454545

 0
 0
 0
 0

 0
 0
 0
 14.5454545454545

 0
 0
 0
 0

 0
 0
 0
 14.5454545454545

 0
 0
 0
 0

 0
 0
 0
 14.5454545454545

 0
 0
 0
 0

 .489043543684755

 1

 .230414746543779

 1

 .145772594752187

 1

 .145772594752187

 1

 .145772594752187

 1

 8.46421517915922E-02

 1

 8.46421517915922E-02

 1

 8.46421517915922E-02

 1

 .178688987115584

 1

 .178688987115584

 1

 8.46421517915922E-02

 1

 9.40468353239913E-02

 1

 9.40468353239913E-02

 1

 7.99398100253926E-02

 1

 7.99398100253926E-02

 1

 7.99398100253926E-02

 1

 7.99398100253926E-02

 1

 6.58327847267939E-02

 1

 6.58327847267939E-02

 1

 6.58327847267939E-02

 1

 6.58327847267939E-02

 1

 6.58327847267939E-02

 1

 .032916392363397

 1

 .032916392363397

 1

 .032916392363397

 1

 .032916392363397

 1

 .032916392363397

 1

 42.7583936800527
 .239938924637365

 1
 1

 42.7583936800527
 .239938924637365

 1
 1

 42.7583936800527
 .239938924637365

 1
 1

 42.7583936800527
 .239938924637365

 1
 1

 1.57528449167686
 2.18126295124877
 2.38268903476781
 25.4545454545455

 .892537313432836
 1
 .744897959183674
 0

 1.0580268973949
 .796160977205802
 .243131534159981

 1
 1
 1

 1.0580268973949
 .796160977205802
 .243131534159981

 1
 1
 1

 1.0580268973949
 .796160977205802
 .243131534159981

 1
 1
 1

 1.0580268973949
 .796160977205802
 .243131534159981

 1
 1
 1

 1.0580268973949
 .796160977205802
 .243131534159981

 1
 1
 1

 .192796012414182
 .130875777074926
 .194505227327984

 1
 1
 0

 .192796012414182

 1

 .192796012414182

 1

 .192796012414182

 1

 .192796012414182

 1

 0
 .119969462318683

 0
 1

 0
 .119969462318683

 0
 1

 0
 .119969462318683

 0
 1

 0
 .119969462318683

 0
 1

 0
 1.09063147562439E-02
 .194505227327984

 0
 1
 0

 0
 1.09063147562439E-02
 .194505227327984

 0
 1
 0

 0
 1.09063147562439E-02
 .194505227327984

 0
 1
 0

 0
 1.09063147562439E-02
 .194505227327984

 0
 1
 0

 .032916392363397
 .327189442687316
 .145878920495988

 1
 1
 1

 .032916392363397
 .327189442687316
 .145878920495988

 1
 1
 1

 .032916392363397
 .327189442687316
 .145878920495988

 1
 1
 1

 .032916392363397
 .327189442687316
 .145878920495988

 1
 1
 1

 .032916392363397
 .327189442687316
 .145878920495988

 1
 1
 1

 .282140505971974
 7.63442032937071E-02
 .243131534159981

 .433333333333333
 1
 0

 7.99398100253926E-02

-1

 7.99398100253926E-02

-1

 7.99398100253926E-02

-1

 7.99398100253926E-02

-1

 .117558544154989

 1

 .117558544154989

 1

 .117558544154989

 1

 .117558544154989

 1

 4.70234176619957E-02

 1

 4.70234176619957E-02

 1

 4.70234176619957E-02

 1

 4.70234176619957E-02

 1

 1.88093670647983E-02
 0
 .243131534159981

 1
 1
 0

 1.88093670647983E-02

 1

 1.88093670647983E-02

 1

 1.88093670647983E-02

 1

 0
 0
 .243131534159981

 0
 0
 0

 0
 0
 .243131534159981

 0
 0
 0

 0
 0
 .243131534159981

 0
 0
 0

 1.88093670647983E-02

 1

 1.88093670647983E-02

 1

 1.88093670647983E-02

 1

 1.88093670647983E-02

 1

 0
 7.63442032937071E-02

 0
 1

 0
 7.63442032937071E-02

 0
 1

 0
 7.63442032937071E-02

 0
 1

 0
 7.63442032937071E-02

 0
 1

 9.40468353239913E-03
 0
 0
 25.4545454545455

 0
 0
 0
 0

 9.40468353239913E-03
 0
 0
 25.4545454545455

 0
 0
 0
 0

 9.40468353239913E-03
 0
 0
 25.4545454545455

 0
 0
 0
 0

 9.40468353239913E-03
 0
 0
 25.4545454545455

 0
 0
 0
 0

 9.40468353239913E-03

 0

 0
 0
 0
 25.4545454545455

 0
 0
 0
 0

 0
 .850692550987021
 1.38584974471189

 0
 1
 1

 0
 .850692550987021
 1.38584974471189

 0
 1
 1

 0
 .850692550987021
 1.38584974471189

 0
 1
 1

 0
 .850692550987021
 1.38584974471189

 0
 1
 1

 0
 .850692550987021
 1.38584974471189

 0
 1
 1

 0
 0
 .170192073911986

 0
 0
 0

 0
 0
 .170192073911986

 0
 0
 0

 0
 0
 .170192073911986

 0
 0
 0

 0
 0
 .170192073911986

 0
 0
 0

 0
 0
 .170192073911986

 0
 0
 0

 1.02040816326531
 .229032609881121
 .218818380743982

 1
 1
 1

 1.02040816326531
 .229032609881121
 .218818380743982

 1
 1
 1

 1.02040816326531
 .229032609881121
 .218818380743982

 1
 1
 1

 1.02040816326531
 .229032609881121
 .218818380743982

 1
 1
 1

 1.02040816326531
 .229032609881121
 .218818380743982

 1
 1
 1

 1.02040816326531
 .229032609881121
 .218818380743982

 1
 1
 1

 4.23210758957961E-02

 1

 4.23210758957961E-02

 1

 4.23210758957961E-02

 1

 4.23210758957961E-02

 1

 4.23210758957961E-02

 1

 4.23210758957961E-02

 1

 7.99398100253926E-02

-1

 7.99398100253926E-02

-1

 7.99398100253926E-02

-1

 7.99398100253926E-02

-1

 7.99398100253926E-02

-1

 7.99398100253926E-02

-1
