# Supplementary material for: Culex quinquefasciatus Holobiont: A Fungal Metagenomic Approach
Source: Front Fungal Biol. 2022 Aug 2;3:918052. doi: 10.3389/ffunb.2022.918052 (PMC10512223; doi:10.3389/ffunb.2022.918052)
Supplement: Supplementary file 6 [file Table_3.docx]

**Supplementary Table 3. Sorensen index (0-1) indicating similarity in fungal communities between *Cx. quinquefasciatus* groups.** L: larvae, M: sucrose-fed male, SF: sucrose-fed females, BF: blood-fed females.

|  | **SF** | **L** | **M** |
| --- | --- | --- | --- |
| **L** | 0.35 |  |  |
| **M** | 0.63 | 0.41 |  |
| **BF** | 0.19 | 0.13 | 0.22 |
